# Mesoporous Nano-Silica Serves as the Degradation Inhibitor in Polymer Dielectrics

**DOI:** 10.1038/srep28749

**Published:** 2016-06-24

**Authors:** Yang Yang, Jun Hu, Jinliang He

**Affiliations:** 1 State Key Laboratory of Power System, Department of Electrical Engineering, Tsinghua University, Beijing 100084, China

## Abstract

A new generation of nano-additives for robust high performance nanodielectrics is proposed. It is demonstrated for the first time that mesoporous material could act as “degradation inhibitor” for polymer dielectrics to sequestrate the electrical degradation products then restrain the electrical aging process especially under high temperature conditions, which is superior to the existing additives of nanodielectrics except further increasing the dielectric strength. Polyethylenimine (PEI) loaded nano-scaled mesoporous silica MCM-41 (nano-MS) is doped into the dielectric matrix to prepare the PP/MCM-41-PEI nanocomposites. PEI provides the amines to capture the electrical degradation products while the MCM-41 brackets afford large adsorption surface, bring down the activating temperature of the absorbent then enhance the absorptive capacity. The electrical aging tests confirm the contribution of the mesoporous structure to electrical aging resistance and FT-IR analysis of the electrical degraded regions demonstrates the chemical absorption especially under high temperature conditions. Take the experimental data as examples, extending the aging durability and dielectric strength of polymer dielectrics by 5 times and 16%, respectively, can have substantial commercial significance in energy storage, power electronics and power transmission areas.

The performance of polymer dielectrics, determines the lifetime and stability of electrical and electronic devices, such as batteries^1^, microelectronics^2^, capacitors^3^,^4^, and cables. Long term electrical degradation of dielectrics is unavoidable to be the main initiators leading to the premature failure of electrical and electronic devices, such as high voltage storage capacitors, cables^5^, etc. especially under high temperature working conditions. In this research, it is demonstrated for the first time that mesoporous material could act as “degradation inhibitor” for polymer dielectrics, i.e., additives that absorb and sequestrate the electrical degradation products then restrain the electrical aging process except further increasing the dielectric strength which is superior to the existing additives of nanocomposites dielectrics (nanodielectrics). This new generation of nano-additives has been proposed here to replace the traditional solid nanoparticles for robust high performance polymer dielectrics and greatly improve the durability and stability of all electronic and electrical devices.

Loading additives to enhance the performance of polymer dielectrics has attracted widespread attention^6^,^7^,^8^. Regarding the predecessor of nanocomposite, polymer/micro-filler composites, large amount (about 50 wt.%) of micron-sized dopants have to be filled into matrix to achieve similar performance improvement brought by little (few wt.%) nano-sized fillers^9^. In addition, high doping concentration of micro-fillers usually brings about increased dielectric loss, decreased breakdown strength, etc.^9^. Thus the micro-fillers are replaced by nano-fillers and the general additives are metallic oxide or ceramic nanoparticles. Because of the large specific surface area and many other novel properties, nano-dopants introduce large amount of phase interface regions into the nanocomposites, which contribute to the enhanced performance of nanocomposite dielectrics^4^,^6^,^10^. Solid nanoparticle additives with different shapes (sphere^11^, rod-like^12^, lamellar^13^, etc.) were applied to increase the phase interface regions then enhance the electrical properties.

Except for the improved dielectric strength, the long term electrical durability of dielectrics is of great significance in improving the lifetime of electrical and electronic devices. Usually, current researches about newly emerging nanodielectrics focused on the breakdown strength^14^, dielectric response^15^, space charge^16^, conductivity and many other dielectric properties, and few have paid attention to the long-term electrical aging process especially under practical temperature conditions. The traditional additives always make no difference in restraining the physical and chemical degradation in electrical aging process. A recent work proposed the “voltage stabilizers” (fullerenes) and has drawn public attention to the long-term electrical endurance of polymer insulation^17^, which contributed to the inhibition of electrical tree inception, and how to restrain the propagation of these fractal micro-cracks, once the electrical trees have been formed, is still an open issue.

Generally, the electrical degradation of polymer dielectrics is accompanied with the generation of small molecular catabolites, which accumulate in the micro-cracks then intensify the local stress (electrical and mechanical) and act as the “degradation accelerator”^5^. It is believed that if the catabolites are removed or sequestrated from the micro-cracks, the electrical degradation process will be greatly restrained. Regarding the sequestration of small molecules, mesoporous materials (including mesoporous silica, mesoporous silicates etc.) have been intensively studied in many related fields of science and technology including catalysis^18^,^19^, gas separation/storage^20^, biomedicine^21^,^22^, and functional assemblies^23^, considering their high specific surface area, good chemical stability, controllable nanoarchitectonics and morphology^24^,^25^,^26^, and other unprecedented intrinsic features. Currently, nano-sized mesoporous particles have been applied to improve the mechanical performance of polymers^27^, and mesoporous structures have been recognized as outstanding electrode materials of electrochemical capacitors for energy-storage applications^28^.

Here, a new promising potential application of mesoporous materials is proposed that nano-scaled mesoporous silica (nano-MS) could act as “degradation inhibitor” for polymer dielectrics, which absorb and sequestrate the catabolites then restrain the electrical degradation process. Essentially superior to the aforementioned traditional additives, mesoporous silica can be loaded with specific functional species and introduce chemical inhibition mechanisms of electrical degradation. Considering that CO_2_ is one of the main electrical degradation products of polymer dielectrics^29^,^30^, the nano-MSs additives are loaded with polyethylenimine (PEI), an excellent long-chain polymer absorbent of CO_2_ with abundant amine groups^31^, to achieve stable chemical sequestration of degradation products and further inhibit the electrical degradation process especially at high temperature for the application of high power density and extreme operating conditions. This research provides a strategy to improve the durability and reliability of polymer dielectrics and extend the lifetime of electrical and electronic devices. The proposed “degradation inhibitor” shall find wide application in electronic and electrical energy areas.

## Results and Discussion

Considering the well-organized mesoporous structure and the pore size of the MCM-41, as is characterized by transmission electron microscopy (TEM) and Barrett-Joyner-Halenda (BJH) analysis, the absorbent PEI of certain molecular weight (Mw ~ 600) was selected. Fourier transform infrared spectroscopy (FT-IR) and the N_2_ adsorption/desorption tests of MCM-41 and MCM-41-PEI were carried out to demonstrate the loading of PEI. The chemical inhibition mechanisms of MCM-41-PEI is believed to improve the electrical degradation resistance under high temperature working conditions. Thus the temperature dependent absorption capacity of MCM-41-PEI was characterized by thermal gravity analysis (TGA) under CO_2_ atmosphere. The electrical tree tests under room temperature and high temperature conditions indicated the high electrical degradation resistance of the proposed “degradation inhibitor” and the chemical inhibition mechanisms were demonstrated by the local FT-IR analysis of the degraded regions.

### Characterization of MCM-41-PEI and Sample Preparation

**Figure 1:**
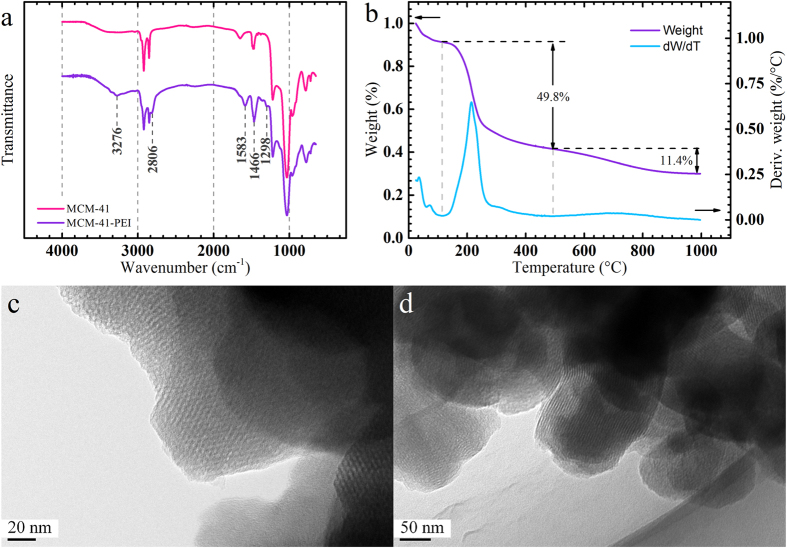
(**a**) FT-IR spectra of the MCM-41 and MCM-41-PEI nanoparticles. (**b**) TGA curve demonstrates that 49.8 wt.% of PEI is loaded into the MCM-41 nanoparticles. TEM images of the MCM-41-PEI nanoparticles. (**a**) The hexagonal well-organized mesoporous structure. (**b**) The image taken in the parallel orientation with mesoporous channels.

Mesoporous silica MCM-41 nanoparticles were modified with PEI by wet impregnation method (see Methods Section) to improve the CO_2_ absorptivity and achieve stable chemical sequestration especially under high temperature conditions^31^. According to some recent investigations, the highest CO_2_ adsorption capacity could be reached at 50 weight percentage (wt.%) loading of PEI in MCM-41-PEI^31^. FT-IR and TGA tests of the MCM-41-PEI nanoparticles were carried out to demonstrate the 50 wt.% loading capacity of PEI into the MCM-41 nanoparticles(see Methods Section). The FT-IR spectrums of MCM-41 and MCM-41-PEI are shown in Fig. 1a. Distinguished from the curve of MCM-41, the IR transmission peaks at 1466 cm^−1^ and 1583 cm^−1^ of MCM-41-PEI represent the C-N stretching vibration and N-H bending vibration of amine groups from PEI. The spectral band near 3276 cm^−1^ represents the C-N stretching vibration of secondary amine in PEI. The asymmetric and symmetric (2806 cm^−1^) stretching vibration bands of the methylene from PEI would overlap with the characteristic peaks of MCM-41. A small peak at 1298 cm^−1^ in the modified sample’s spectrum can be attributed to the wagging vibration of the methylene from PEI which cannot found in MCM-41. The loading capacity is assessed by TGA tests. As is shown in Fig. 1b, the weight loss of organic moieties (100 °C ~ 500 °C) is 49.8% which confirms the 50 wt.% loading capacity. The 11.4% weight loss above 500 °C is attributed to the residue template in MCM-41.

The particle size of the nano-MCM-41-PEI is about 200 nm (see Supporting Information, Supplementary Note 1) and the mesoporous structure is characterized by TEM in Fig. 1. The BJH pore size distribution, pore volume and BET surface area (see Supporting Information, Supplementary Note 2) of nano-MCM-41 before and after loading PEI are obtained from the N_2_ adsorption/desorption tests (Methods Section). The BJH adsorption/desorption cumulative pore volumes are 0.2768/0.2087 cm^3^/g and 0.0404/0.0332 cm^3^/g before and after loading PEI. It can be assumed that 50 wt.% loading of PEI can completely fill the mesoporous pore channels of MCM-41.

MCM-41-PEI nanoparticles were doped into polypropylene random copolymer (PP-R) by melt blending method to obtain the 0.5 wt.% PP/MCM-41-PEI nanocomposites (see Methods Section). In order to demonstrate the contribution of mesoporous structure, solid silica nanoparticles of similar size (200–300 nm) were doped into PP-R by the same method to prepare the PP/SiO_2_ nanocomposites and the control samples. PP/SiO_2_ nanocomposites with the same wt.% and particle volume percentage (vol.%) of silica as the PP/MCM-41-PEI nanocomposites were prepared. According to the cumulative pore volume of MCM-41 nanoparticles obtained from BJH adsorption/desorption tests (about 0.3 cm^3^/g) and the solid density of silica (about 2.2 g/cm^3^), 0.83 wt.% PP/SiO_2_ nanocomposites would theoretically contain the equivalent vol.% of nanoparticles as the 0.5 wt.% PP/MCM-41-PEI. All the nanocomposites together with the polymer matrix (PP-R) were hot-pressed into cubic samples with a steel needle electrode pre-embedded (see details in Methods Section) for the following electrical aging tests. The film samples were also prepared by hot-pressing method for electrical breakdown tests.

### Electrical Degradation Tests

The electrical degradation (electrical tree) resistance can be directly assessed by the duration of various degradation stages (including tree inception, propagation and branching) under the same aging conditions (electrical aging test I) or the electrical tree size after the same aging period (electrical tree test II). Thus the aforementioned electrical tree test I was carried out at room temperature while the electrical tree test II was performed at high temperature (see Methods Section and Supporting Information, Supplementary Note 3).

**Figure 2:**
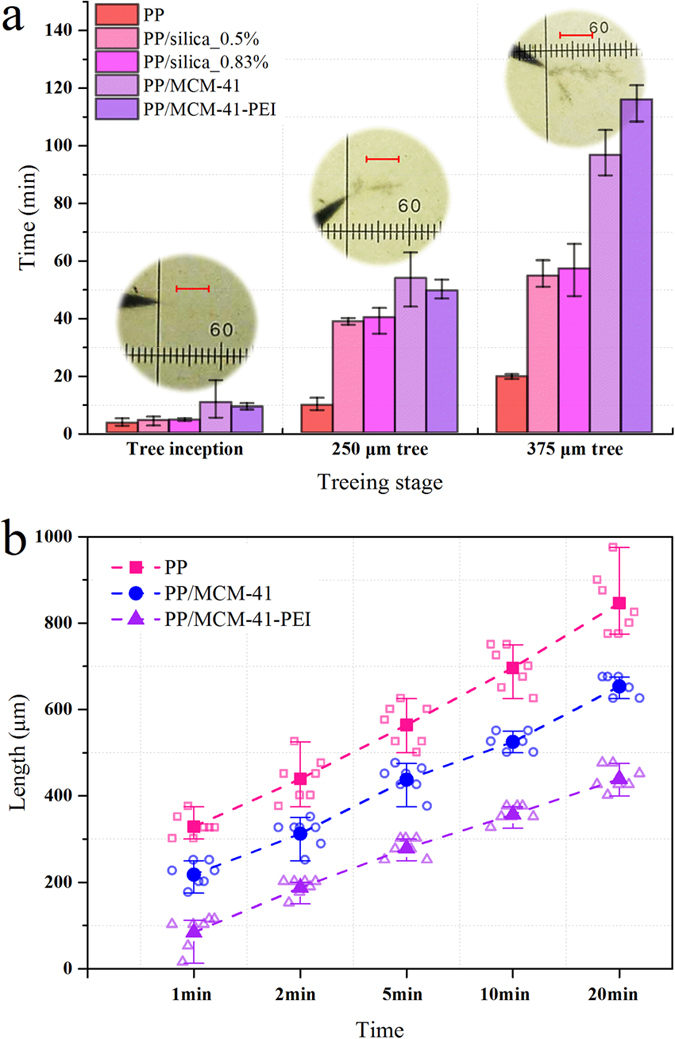
Electrical tree tests. (**a**) The average duration of each degradation stages in electrical tree test I. All the parallel experiments are repeated five times with the maximum and minimum results removed to get the error bar data. The scale bars in the optical images represent 125 μm. (**b**) The average electrical tree size after the voltage has been applied for 1, 2, 5, 10, and 20 minutes at 80 °C and all the parallel experiments were repeated 10 times, 7 results were selected to obtain the data points and error bar in electrical tree test II.

The average duration of each degradation stages in test I are shown in Fig. 2a. The average tree inception times of PP/nano-MS are higher than twice of PP’s and the propagation time of PP/nano-MS before the trees reach 375 μm is 5 times longer than that of PP. Regarding the PP/SiO_2_ nanocomposites, representing the traditional polymer/solid ceramic nanocomposites, the solid silica nanoparticles enhance the electrical degradation resistance but the improvement is not as remarkable as the nano-MSs. The nano-MSs provide much higher electrical degradation resistance than solid SiO_2_ especially during the propagation stage of the electrical tree. There is no significant difference between PP/MCM-41 and PP/MCM-41-PEI in electrical tree test I because the organic amine PEI is believed to exhibit high reaction activity with the degradation products at high temperature^31^. Considering the operating temperature of many dielectric applications including power transmission cables (60–100 °C)^32^, the electrical tree test II is designed under 80 °C conditions and the results are shown in Fig. 2b.

The electrical degradation degree of PP/nano-MS nanocomposites after the same time of electrical aging is still lower than PP at 80 °C. Distinguished from electrical tree test I, the PP/MCM-41-PEI nanocomposites exhibit much higher electrical degradation resistance than PP/MCM-41 and exert nearly half of the PP’s degradation degree in test II (Fig. 2b). The PP/MCM-41 nanocomposites lose the resistance of electrical degradation under high temperature condition (80 °C). This difference between PP/MCM-41 and PP/MCM-41-PEI can be attributed to the physical absorption ability decline of MCM-41 and chemical sequestration ability improvement of PEI at high temperature which will be demonstrated below.

### Demonstrations of the Chemical Absorption

**Figure 3:**
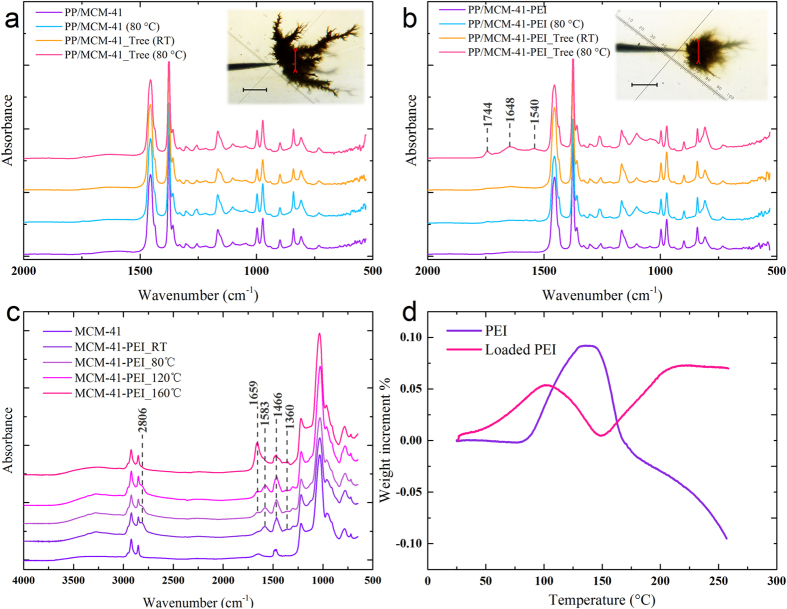
FT-IR analysis of electrical tree regions of (**a**) PP/MCM-41 nanocomposites and (**b**) PP/MCM-41-PEI nanocomposites at RT and 80 °C. The FT-IR spectrums of unaged regions were presented for comparison. (**c**) MCM-41-PEI nanoparticles treated in air were tested to confirm the temperature dependence of chemical absorption. The red lines in the optical images highlight the analyzed regions of the electrical tree and the scale bars represent 500 μm. (**d**) The weight increment percentage curves of pure PEI and loaded PEI calculated from the TGA analysis of MCM-41, PEI and MCM-4-PEI under CO_2_ atmosphere.

Electrical degradation is a complex aging process accompanied with various chemical degradation including thermal degradation^33^, oxidation^30^, field ionization, photodegradation, hydrolysis etc.^5^. The degradation process greatly depends on the oxygen in the free volume of the polymer which is responsible for the oxide products (CO_2_, H_2_O etc.)^34^. After the electrical tree is initialed, the small molecular degraded products would accumulate in the micro-sized tree channels and lead to local stress enhancement (mechanical and electrical) and partial discharge then intensity the degradation process. The electrical degradation resistance enhancement of PP/nano-MS nanocomposites in electrical tree test I and II would be attributed to the physical (MCM-41) and chemical (PEI) absorption of the degraded products which are greatly influenced by the temperature conditions. Local FT-IR analysis was performed in the electrical tree regions of long-term (more than 30 h) aged samples (Fig. 3). In order to confirm the temperature dependence of chemical absorption, the FT-IR tests of MCM-41-PEI nanoparticles were carried out after the samples have been treated at room temperature (RT), 80 °C, 120 °C and 160 °C for 2 h in air.

As is shown in Fig. 3a, there is nearly no difference between the curves of degraded and original regions in PP/MCM-41 nanocomposites. The absorbed CO_2_ and H_2_O molecules cannot be characterized by FT-IR because of the subtraction of background and this physical absorption would not produce any specific products. Considering that most of the mesoporous channels of MCM-41-PEI have been filled by 50 wt.% of PEI, the chemical absorption of CO_2_ and H_2_O take the dominant role in PP/MCM-41-PEI nanocomposites. Regarding the sample aged at 80 °C (Fig. 3b), the absorption peak at 1540 cm^−1^ indicates the formation of primary amine salts (symmetric angular vibration of NH_3_^+^). The absorption peaks near 1648 cm^−1^ present the stretching vibration of C=O in amide bond. The absorption peak at 1740 cm^−1^ may be attributed to the absorbed water by the organic amine^34^. Figure 3c confirms the temperature-dependent absorption of MCM-41-PEI. The absorption band near 1659 cm^−1^ is unclear considering the complex reactions with other species in air and two interpretations are proposed: i) the blue shift of absorbed water band near 1548 cm^−1^ caused by interaction with some species^35^, or ii) liberation of some molecular interaction under high temperature condition. The formation of amide (C=O bond) is activated above 80 °C and the characteristic band of HCO_3_^−^ in the range of 1360–1350 cm^−1^ is observed^35^. This absorption band would overlap with the characteristic peaks of PP in Fig. 3b for the samples aged at room temperature. As the temperature rises to 160 °C, the characteristic peaks of the methylene (2806 cm^−1^) and amine groups (1583 and 1466 cm^−1^) in MCM-41-PEI recede to the same as the MCM-41 sample which indicates that PEI would be lost. Further demonstration of the temperature dependence and the losing of PEI at very high temperature are shown in the TGA analysis under CO_2_ atmosphere.

TGA tests of MCM-41, PEI and MCM-41-PEI are carried out under CO_2_ atmosphere from the room temperature to 250 °C (see Methods Section). The weight increment percentage of MCM-41 is subtracted from MCM-41-PEI to obtain the weight increment percentage of loaded PEI. As is shown in Fig. 3d, the pure PEI starts to absorb CO_2_ above 80 °C and the absorption peak is located at about 100 °C. After the temperature rise above 160 °C, the weight return to the initial value and continue to decrease. It is assumed that the CO_2_ molecules desorb from the organic amine and the PEI would start to gasify or degrade above 160 °C which agrees well with the FT-IR spectroscopy of the heat treated MCM-41-PEI nanoparticles. The experimental results demonstrate that the activating temperature of pure PEI is above 80 °C which greatly limits its direct application as the degradation inhibitor in power cable and many other polymer dielectrics. The weight increment percentage curve of loaded PEI indicates that the MCM-41 brackets bring down the activating temperature of PEI to 50 °C or lower. The absorption peak of loaded PEI is observed at about 100 °C which is much lower than the pure PEI. It can be concluded that the porous silica MCM-41 provide PEI with large reaction area and bring down the start and peak temperature of absorption then greatly improve the absorption ability of PEI under the operating conditions of power cable (60~100 °C) and many other polymer dielectric applications. However, the direct CO_2_ absorption of MCM-41-PEI is limited under room temperature conditions which results in the different chemical absorption mechanisms in electrical tree test I and II.

### Physical and Chemical Inhibition Mechanisms of Electrical Degradation

**Figure 4:**
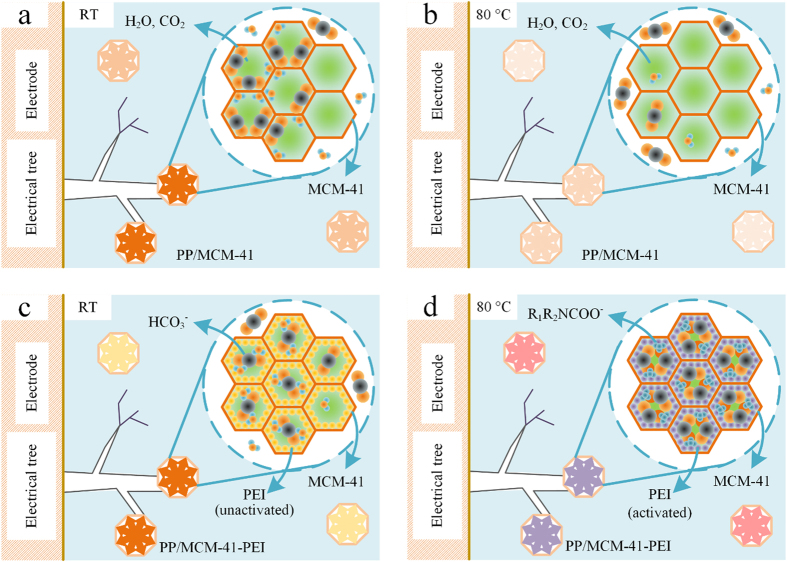
Schematics of different CO_2_ absorption mechanisms. (**a**) Physical absorption in PP/MCM-41 at RT. (**b**) Declined physical absorption in PP/MCM-41 at 80 °C (**c**) Chemical absorption in PP/MCM-41-PEI at RT and bicarbonate is produced under the catalysis of amine. (**d**) Enhanced chemical absorption in PP/MCM-41-PEI at 80 °C and the carbamate-formation reaction is activated.

The different CO_2_ (together with H_2_O) absorption mechanisms of PP/MCM-41 and PP/MCM-41-PEI under room temperature and 80 °C conditions are illustrated in Fig. 4 and interpreted below.

In general, the physical absorption is based on the BET (Brunauer-Emmett-Teller) multi-molecular absorption model, which describes the equilibrium absorption on solid below the saturated vapor pressure of the gas:^36^


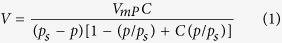


where *V* is the volume of absorbed gas under equilibrium pressure *p*, *V*_m_ is volume of the first layer of absorbed gas molecules that cover the surface of solid absorbent, *p*_s_ is the saturated vapor pressure of the gas and *C* is a constant.

The BET theory attributes the multi-molecular absorption to the similar thermodynamic process as liquidation. However, in theory, the gas molecular cannot be liquefied when the temperature is above its critical temperature (supercritical gas). The saturated vapor pressure of supercritical gas can be treated as infinity and the absorption of supercritical gas under normal pressure conditions would be negligible according to Equation (1). Researches indicate that the absorption of supercritical gases on solid surface is very weak and evident surface absorption could only be observed under very high pressure^37^. Regarding carbon dioxide, the critical temperature is 31.26 °C, a little higher than the sample temperature in electrical tree test I (about 20 °C). As the temperature rise beyond 31.26 °C in electrical tree test II (80 °C), the degradation product CO_2_ behaves as supercritical gas and the CO_2_ absorption ability of MCM-41 would be greatly reduced. Thus it is believed that the mesoporous silica in PP/MCM-41 nanocomposites could absorb the electrical degradation products and enhance the electrical tree resistance at room temperature as illustrated in Fig. 4a. Under high temperature conditions, the mesoporous silica loses its physical absorption ability (Fig. 4b) and the electrical tree resistance improvement of PP/MCM-41 in electrical tree test II is receded.

The chemical absorption of MCM-41-PEI should be attributed to the organic amine absorbent PEI. FT-IR spectroscopy indicates that PEI contains three kinds of amine groups including primary amine, secondary amine and tertiary amine (see Supporting Information, Supplementary Note 4). According to the zwitter-ion mechanism proposed by recent studies^34^,^38^, the absorption of CO_2_ by primary amine and secondary amine can be realized by the following reactions:


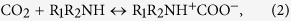



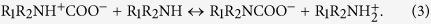


The amine first combines with CO_2_ to form zwitter-ion then the proton-transfer reaction with another amine produces carbamate and amine salts, this agrees well with the aforementioned FT-IR tests. Tertiary amine, without any hydrogen atom bonded with the nitrogen atom, has difficulty in reacting with CO_2_ directly^39^. Apart from the above mentioned fast direct chemical reactions between the amine groups and CO_2_, all three kinds of amine could react with bicarbonate considering the participation of H_2_O and the reaction between CO_2_ and H_2_O will be catalyzed by amine to product bicarbonate which has been demonstrated in the FT-IR tests of MCM-41-PEI nanoparticles:^38^,^40^


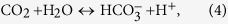



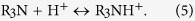


Generally, the formation of bicarbonate is slow and controls the overall reaction with CO_2_ in Equations (4) and (5)^35^. The direct CO_2_ absorption of PEI, illustrated in Equations (2) and (3), can be activated only at high temperature. The temperature dependence of the direct CO_2_ absorption of PEI is further demonstrated by TGA tests under CO_2_ atmosphere. The CO_2_ TGA curves indicate that the absorbability of pure PEI is activated above 80 °C, which greatly limits its direct application as the degradation inhibitor in many polymer dielectrics. Regarding the MCM-41-PEI, the mesoporous silica brackets bring down the activating temperature and the absorption peak of PEI to 50 °C and 100 °C then improve the absorption ability under the operating conditions of many polymer dielectrics, such as power transmission cable (60–100 °C). Thus the chemical absorption can be summarized as the amine catalyzed hydrolysis of CO_2_ (produce bicarbonate and amine salts) at room temperature (Fig. 4c) and the direct absorption of CO_2_ (produce carbamate and amine salts) at 80 °C (Fig. 4d). The direct chemical absorption of MCM-41-PEI is activated at 80 °C which plays the dominant role and further enhance the electrical tree resistance in electrical tree test II.

## Conclusions

It can be concluded that the difference in electrical tree resistance between PP/MCM-41 and PP/MCM-41-PEI nanocomposites at high temperature is due to the decline of physical absorption and the activation of direct carbamate-forming chemical absorption. Additionally, it is well-known that mesoporous silica MCM-41 and many other inorganics would generate micro-cracks or weak points in the phase interface regions if appropriate surface modification were not performed. Thus, incorporation of the bare MCM-41 will introduce some defects and reduce the expected improvement of degradation resistance especially when the pore size is not suitable for the polymer chain to stretch in. Regarding the MCM-41-PEI, it has been demonstrated that the mesoporous pore channels are completely filled with PEI which not only acts as the absorbent of degradation products but improves the compatibility of PP/MCM-41-PEI blends and restrains the introduction of new defects.

The “degradation inhibitor”, as is first proposed here to enhance the durability and stability of polymer dielectrics, paves the way to easy-to-manufacture low-cost and robust nanodielectrics. The organic amine functionalized mesoporous silica nanoparticles MCM-41-PEI greatly improves the electrical degradation resistance of PP/nano-MS nanocomposites except increasing the electrical strength of the nanocomposites (see Supporting Information, Supplementary Note 5) by more than 16% not only under room temperature conditions but at high temperature, this is of great significance in electronic and electrical energy areas. This research also endows the mesoporous materials with a new promising role to replace the traditional nano-fillers of nanodielectrics.

## Methods

### Materials and Modification

Mesoporous silica MCM-41 was purchased from Real & Lead Chemical Co., Ltd (Tianjin, China) and then modified with PEI (Mw ~ 600, 99%, Aladdin Industrial Inc. China) by wet impregnation method. 0.3 g PEI was dissolved in methanol under stirring for 15 min, then 0.3 g (50 wt.%) MCM-41 nano-MSs were added into the solution and the mixture was stirred under room temperature for 30 min. Finally the slurry was dried at 75 °C for 16 h after cleansed with methanol to remove the residual unutilized PEI and the PEI modified MCM-41 nanoparticles (nano-MCM-41-PEI) were obtained.

### Characterization

FT-IR spectra were carried out on a Nicolet iS10 (Thermo, USA) FT-IR spectrometer in attenuated total reflection (ATR) mode between 650 and 4000 cm^−1^. The samples were analyzed at 0.482 cm^−1^ resolution and 16 scans co-averaged. The background of the atmosphere is measured and subtracted from each spectrum before measuring. TGA (TA Q500, USA) tests were carried out at the heating rate of 10 °C per min under nitrogen atmosphere from the room temperature (about 25 °C) to 800 °C. Transmission electron microscopic (TEM) analysis was performed on a Tecnai G20 (FET, USA) microscope. The nano-MCM-41-PEI particles were first dispersed in ethanol and treated with ultrasound for 15 min at room temperature before the sample preparation. The BJH pore size distribution, pore volume and BET surface area calculation were carried out based on the N_2_ adsorption-desorption isotherms by a TriStar II 3020 (Micromeritics, USA) specific surface area and porosity analyzer. According to the TGA initial organic weight loss temperature of MCM-41-PEI, the samples were pretreated under 100 °C for 4 h to remove the adsorptive. The adsorption-desorption temperature is 77 K.

### Sample Preparation

PP/MCM-41-PEI and the compared PP/SiO_2_ nanocomposites were prepared by melt blending method in an internal mixer under 200 °C. The cubic samples with pre-embedded steel needle electrode were prepared by hot-pressing in a vulcanizing press under 200 °C and sized by a steel mould. A small piece of conductive rubber was penetrated by the needle electrode and embedded at one side of the sample to provide a conductive contact with the external electrode. The tip of the needle electrode was located near the center of the 20 × 15 × 3 mm cubic sample (about 7 mm from the grounding surface). The film samples with different thickness were prepared by hot-pressing under 200 °C in steel mould.

### Electrical degradation tests

In both of the two tests, the samples were clamped between a high voltage plate electrode and a ground plate electrode which were in good contact with the needle electrode and grounding surface of the samples, respectively, and AC high voltage of 30 kV (peak-to-peak value) was applied to the samples.

## Additional Information

**How to cite this article**: Yang, Y. *et al*. Mesoporous Nano-Silica Serves as the Degradation Inhibitor in Polymer Dielectrics. *Sci. Rep.* **6**, 28749; doi: 10.1038/srep28749 (2016).

## Supplementary Material

Supplementary Information
