# Supplementary material for: Mesoporous Nano-Silica Serves as the Degradation Inhibitor in Polymer Dielectrics
Source: Sci Rep. 2016 Jun 24;6:28749. doi: 10.1038/srep28749 (PMC4919646; doi:10.1038/srep28749)
Supplement: Supplementary Information [file srep28749-s1.doc]

# Supplementary Information

**Mesoporous Nano-Silica Serves as the Degradation Inhibitor in Polymer Dielectrics**

Yang Yang, Jun Hu, Jinliang He*

State Key Laboratory of Power System, Department of Electrical Engineering, Tsinghua University, Beijing 100084, China

*E-mail: hejl@tsinghua.edu.cn.

**Supplementary Note 1: TEM images**

**
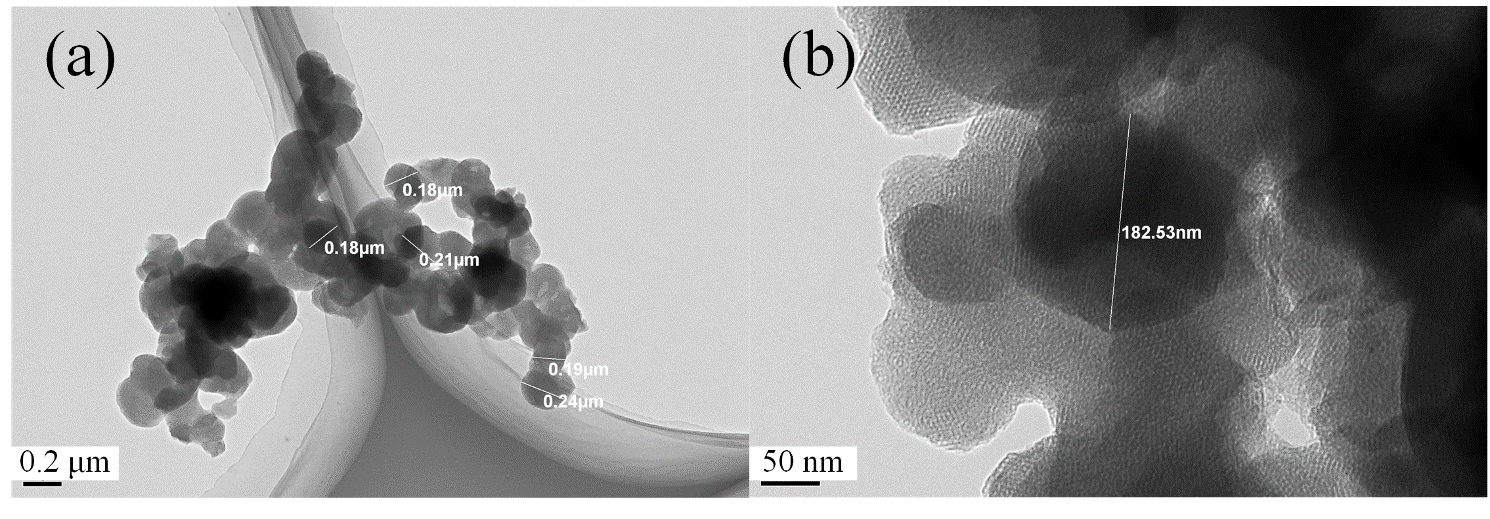
**

**Figure S1.** TEM images of MCM-41-PEI indicate that the particle size can be estimated as 150~250 nm.

**Supplementary Note 2: N_2_ adsorption/desorption tests**

The N_2_ adsorption/desorption tests indicated that the BJH adsorption/desorption average pore sizes of nano-MCM-41 are 2.45/2.25 nm and the most probable pore size is about 2.10 nm. After loading of PEI, the most probable pore size changes to about 2.00 nm and the BJH adsorption/desorption average pore sizes are 2.96/2.90 nm.


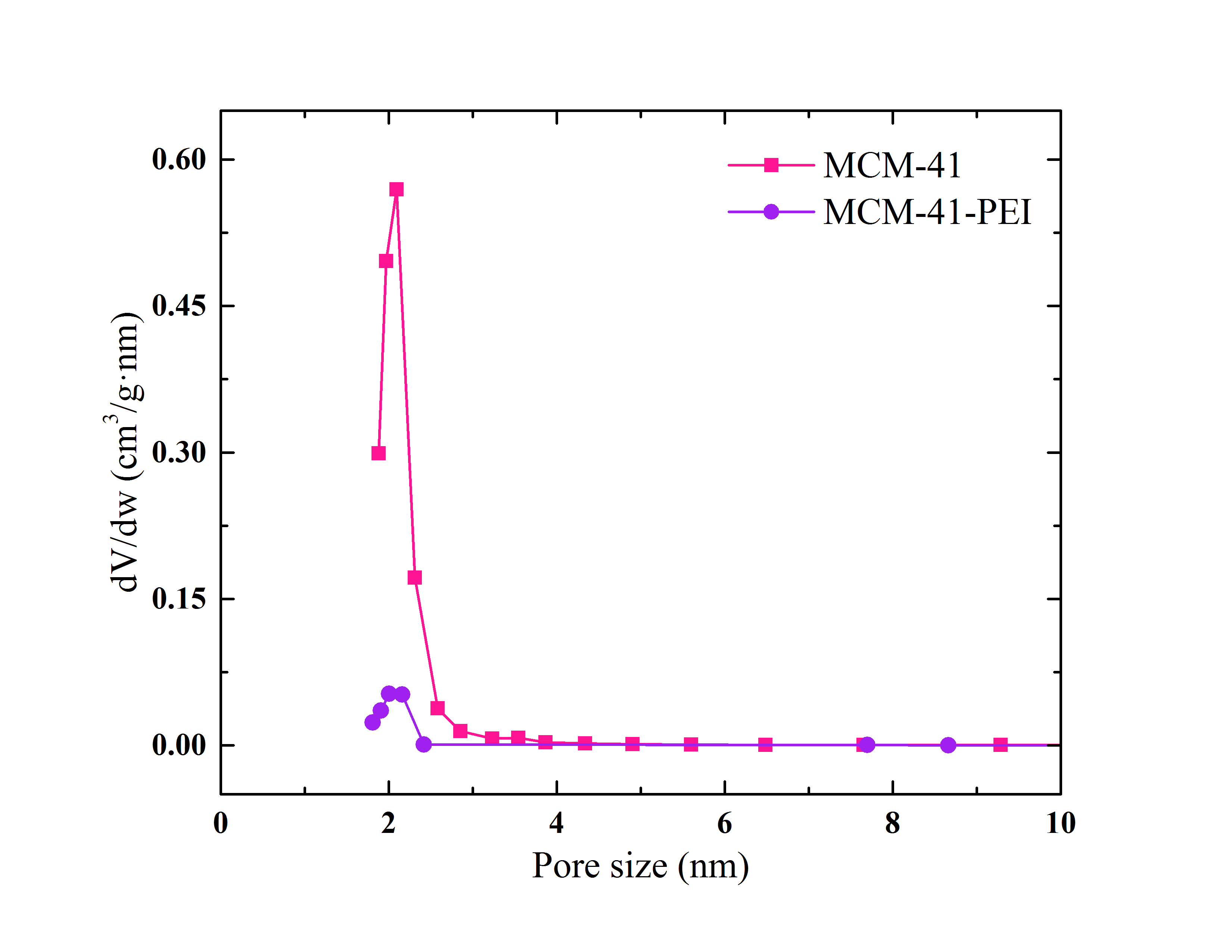


**Figure S2.** The BJH pore size distribution of nano-MCM-41 and nano-MCM-41-PEI obtained from the nitrogen adsorption/desorption isotherms.

**Supplementary Note 3: Electrical tree tests**

The PP and nanocomposite samples with pre-embedded steel needle electrode are prepared by hot-pressing in a vulcanizing press under 200 °C and the sample is illustrated in **Figure S3**a. The diameter of the stainless steel needle electrode is 0.25 mm. The SEM image of the needle tip shows that the tip angle is about 30 ° and the tip radius is about 1 μm. The electrical tree test I and II are carried out with different electrode structures shown in Figure S3b and c, respectively. The sample pool is made of transparent PMMA for the sake of optical microscopic observation. The external plate electrodes are fastened in good contact with the surface of the sample and the high voltage is applied to the HV electrode while the ground electrode is connected to the ground. The samples are soaked in silicone oil during the electrical aging process to avoid surface flashover. The previous experimental results and other researches indicate that electrical tree would go through a relatively slow propagation stage accompanied with tip splitting during branching stage which brings trouble in evaluating the degradation degree. Thus the test I and test II were performed before the electrical trees grow into seriously branched fractal tree channels.

In electrical tree test I, the samples were aged one by one and uninterruptedly monitored with an optical microscope. Limited by the transparency of the samples, a tiny initial electrical tree of 10~20 μm is generally the minimum scale can be observed which is identified as the inception of electrical tree. Two other degradation stages, when the longest electrical tree channel reached 250 μm and 375 μm, were recorded. The aforementioned PP/SiO_2_ nanocomposites with the same wt. % and vol. % of nanoparticles as the PP/nano-MS nanocomposites were tested as the contrastive samples to demonstrate the contribution of mesoporous structure. In electrical tree test II, considering the decelerating tree propagation process, the tree size was recorded after the voltage has been applied for 1, 2, 5, 10, and 20 min.


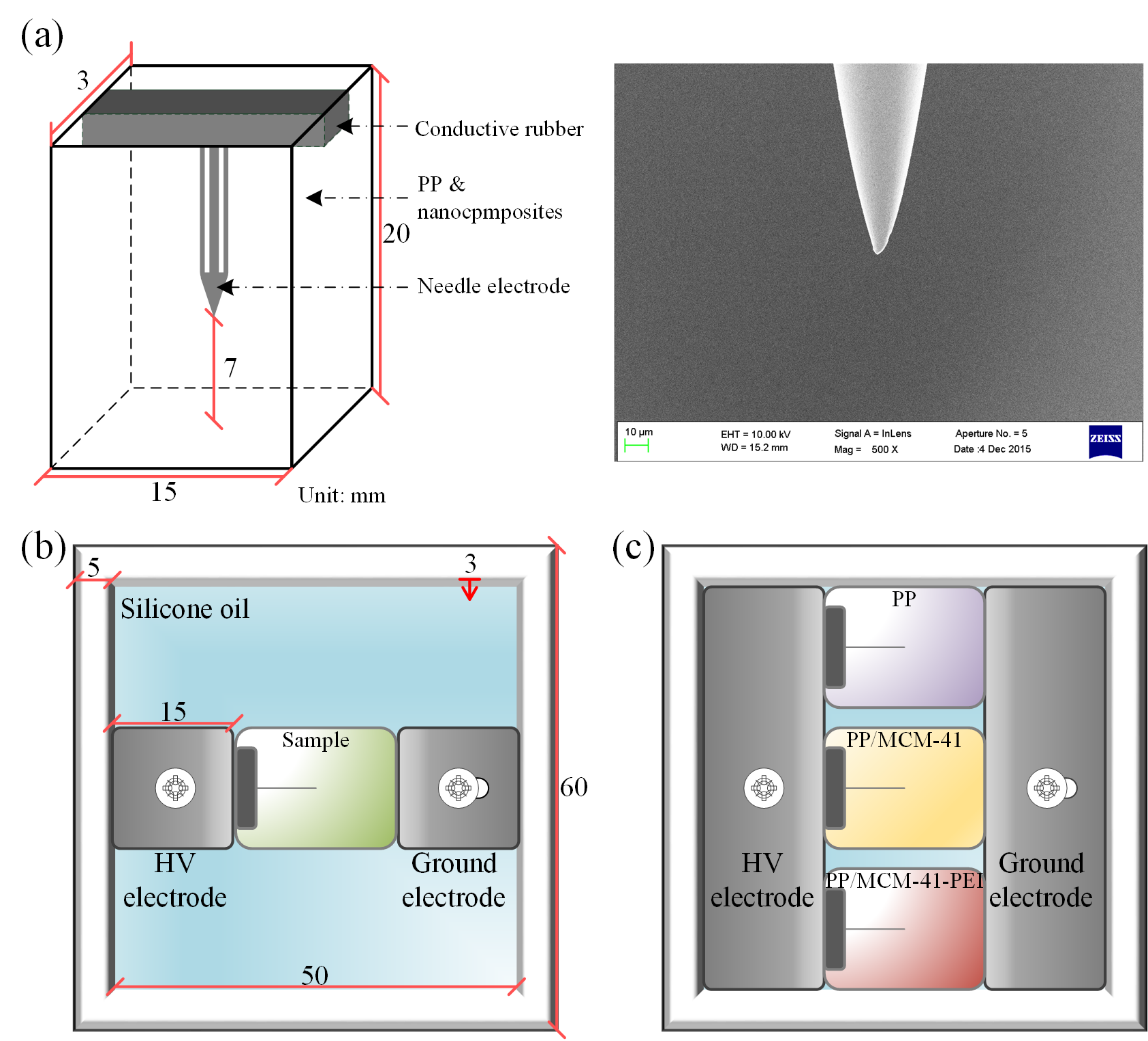


**Figure S3.** (a) Schematic of the sample and SEM micro morphology of the needle electrode. Electrode structures of electrical tree test (b) I and (c) II.

**Supplementary Note 4: FT-IR spectroscopy of PEI**

The FT-IR spectrum curves of PEI is shown in **Figure S4**. In order to reveal the detailed characteristic peaks of the curves, only the spectrum from 650 to 4000 cm^-1^ is presented here. The FT-IR spectrum curve shows the characteristic stretching vibration peaks of C-N bonds at 1045 cm^-1^ (tertiary amine) and 1120 cm^-1^ (primary amine) The transmission peaks near 3348 cm^-1^ and 3280 cm^-1^ represent the stretching vibration of N-H bonds in primary and secondary amine, respectively. In addition, there are some transmission peaks between 910~750 cm^-1^ which are attributed to the twisting vibration band of primary amine. It can be concluded that the PEI contains primary amine, secondary amine and tertiary amine as is illustrated in Figure S4.


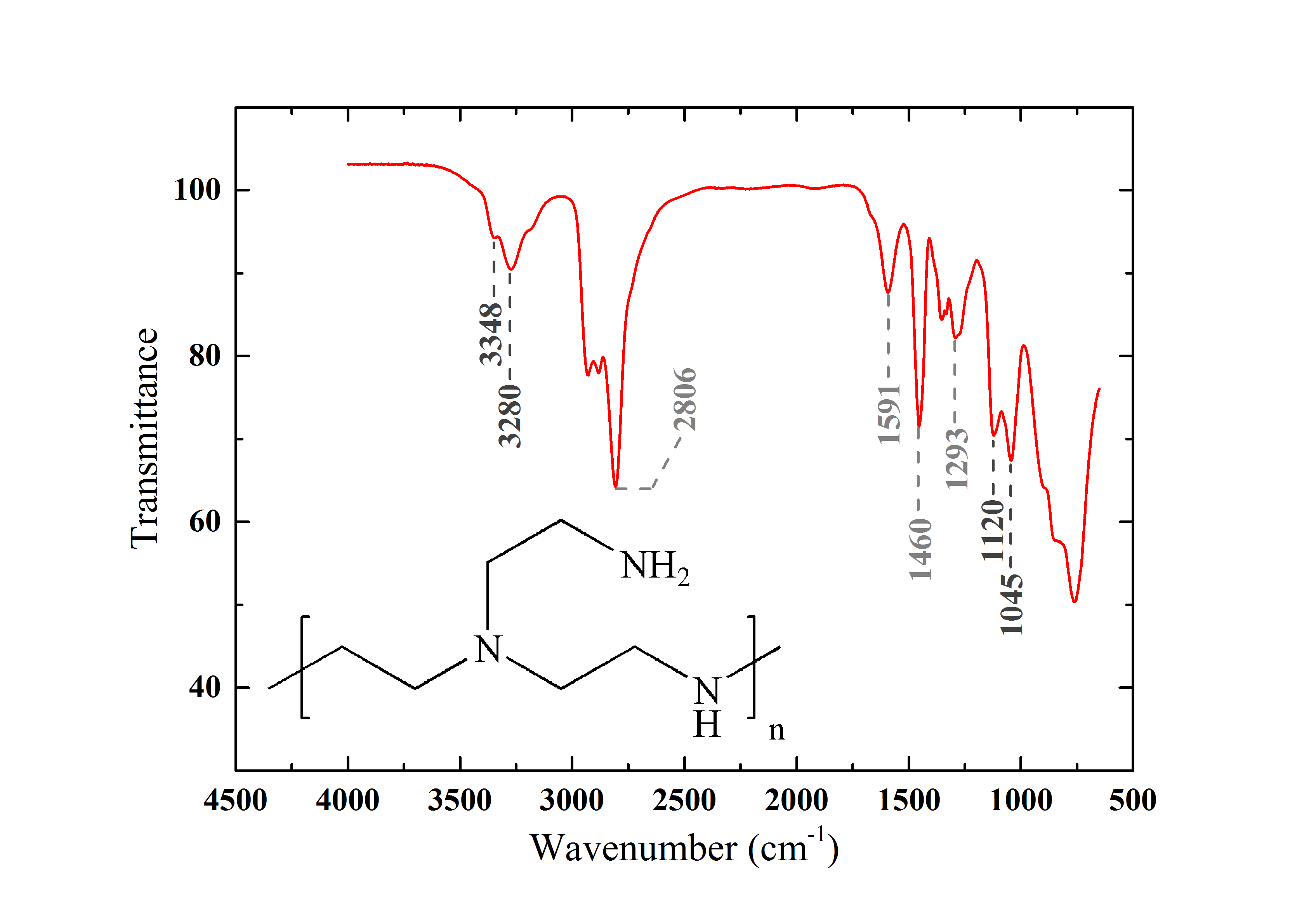


**Figure S4.** FT-IR spectrum of PEI.

**Supplementary Note 5: Direct current (DC) breakdown tests**

In order to demonstrate the dielectric strength of the newly developed PP/nano-MSs nanocomposites, electric breakdown tests under DC voltage are carried out on PP/MCM-41-PEI, PP/MCM-41, PP/SiO2 (0.83 wt.% and 0.5 wt.%) and PP film samples of the thickness about 80 μm. The tests are performed under room temperature condition and the sample films are put between a pair of steel spherical electrodes (10 mm in diameter) according to IEC std. 60243-2: 2001. The sample and the electrodes are soaked in silicone oil to avoid surface flashover. DC voltage of 1 kV/s rising rate is applied to the sample and the breakdown voltage will be recorded to calculate the electric breakdown field. Every sample films are tested on 30 points (parallel tests) and the data points with the bias to average value larger than the standard deviation are treated as invalid. The remaining valid data points are fitted to a Weibull distribution according to IEC std. 62539: 2007. The 2-parameter cumulative Weibull distribution function (equation S1) describes a set of independent statistical events including electric breakdown strength:^S1^

$P(E)=1-\exp[-(\frac{E}{\alpha})^{\beta}]$ (S1)

where *E* is the calculated electric breakdown field, *α* is scale parameter characterizing a central strength value below which the probability of breakdown event is 63.2%, *β* is a shape parameter of Weibull distribution. Then the curves of log[-ln(1-*P*)] versus *E* and the parameters *α*, *β* are shown in **Figure S5**.

The shape parameters *β* of the experimental data points are larger than 10 which indicate narrow distributions of the results. The characteristic breakdown strengths *α* of the five samples indicate that the breakdown strength of PP/MCM-41-PEI is much higher than the PP matrix and the other nanocomposites. The electrical strength decrease of PP/MCM-41 may be caused by the unfilled pores. The electrical strength improvement introduced by the “degradation inhibitor” (more than 16%) is much higher than the traditional SiO_2_ nanoparticles (less than 5%) which is attributed to the increased phase interface regions in PP/MCM-41-PEI nanocomposites. It can be concluded that replacing the traditional additives with the mesoporous nanoparticles will further improve the dielectric strength of the nanocomposites.


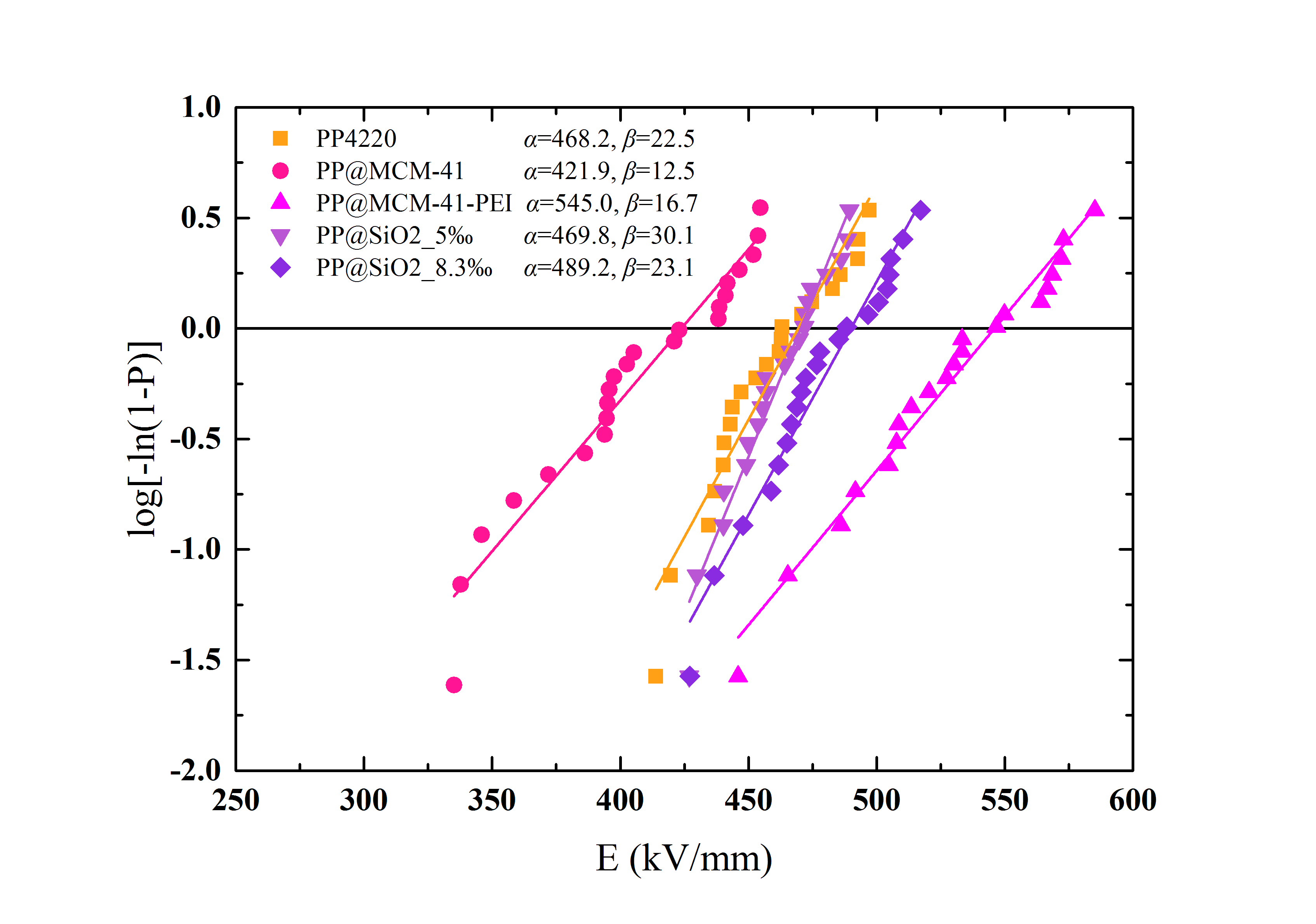


**Figure S5.** Weibull diagram of DC breakdown strength with 1kV/s voltage rate.

**Supplementary References**

S1. A. Laifaoui, M. S. Herzine, Y. Zebboudj, J. Reboul & M. Nedjar, Breakdown strength measurements on cylindrical polyvinyl chloride sheaths under AC and DC voltages. *Ieee Trans. Dielectr. Electr. Insul.* **21,** 2267-2273 (2014).
